# Evaluating Impacts of a One Health Approach to Companion Animal Health and Management in a Remote Aboriginal Community in the Northern Territory, Australia

**DOI:** 10.3390/ani10101790

**Published:** 2020-10-01

**Authors:** Tamara Riley, Raymond Lovett, Joanne Thandrayen, Bonny Cumming, Katherine A. Thurber

**Affiliations:** 1 National Centre for Epidemiology and Population Health, Research School of Population Health, The Australian National University, Canberra, ACT 2600, Australia; raymond.lovett@anu.edu.au (R.L.); joanne.thandrayen@anu.edu.au (J.T.); katherine.thurber@anu.edu.au (K.A.T.); 2 Animal Management in Rural and Remote Indigenous Communities, Darwin, NT 0801, Australia; bonny.cumming@amrric.org

**Keywords:** Aboriginal community, animal health, remote, One Health, dogs, cats, population management

## Abstract

**Simple Summary:**

Many remote Australian Aboriginal communities face barriers in accessing animal health care for their pets. A community that faces these barriers implemented a community-driven program with the objective of improving animal health and population management. The program was evaluated by comparing the percentage of animals desexed, the body and hair condition of dogs, and the presentations at the health clinic for dog bites before versus after 12 months of program implementation. Results show improved animal health measures and no measurable change in human presentations for dog bites. This program, with One Health considerations, could be suitable for other communities facing similar animal health care barriers.

**Abstract:**

This study evaluated a community-driven animal health and management program in the remote community of Wadeye, Northern Territory. This evaluation used a pre-post design to assess changes in animal and human health outcomes over a 12-month period of program implementation, from June 2018 to June 2019. The evaluation assessed the program by comparing animal health outcomes before versus one year after program implementation and comparing human health outcomes before versus during the first 12 months of the program. Outcome measures included the desexing status of dogs and cats, body condition and hair score of dogs, and rates of people presenting to the health clinic for a dog bite. Animal health outcomes significantly improved after program implementation. From pre to post program, there was a 77% increase in the prevalence of good body condition score among dogs and a 9% increase in the prevalence of good hair score among dogs, and the prevalence of desexed dogs and cats more than doubled. There was no significant change in the number of people presenting to the health clinic for a dog bite. Consideration on how to further incorporate human and environmental health aspects into the program could be useful for future One Health programs.

## 1. Introduction

The World Health Organization estimates that 61% of all human pathogens are zoonotic (can be transmitted between animals and people) and 75% of emerging diseases affecting people in the last decade have originated from animals [1]. The World Organisation for Animal Health also suggests that controlling zoonotic pathogens in animals is an effective way of protecting people [2].

Animals have the potential to harbour and spread exotic diseases. The North of Australia is particularly prone to the introduction of exotic diseases due to the vast coastline, remoteness of communities, and the movement of animals and people from neighbouring countries where diseases not yet present in Australia are endemic [3,4]. The implementation and longevity of effective animal health programs in the North of Australia can mitigate overpopulation and improve the health of animals.

This is particularly pertinent in rural and remote Aboriginal communities. People living in rural and remote areas of Australia are more likely to experience greater health concerns and poorer access to health services compared to urban communities [5,6], and this encompasses access to animal health care [7]. Due to this barrier, the poor health of dogs and cats kept as pets can affect human health, particularly where animals and humans live closely together. Large populations of companion animals can lead to increased spread of disease, reduced capacity to prevent disease, and increased risk of physical injury to both animals and humans [7,8]. They can also affect the environment and native wildlife populations through predation, disturbances, and disease transmission [9].

A large proportion of communicable diseases in companion animal populations are easily preventable through access to animal medicines and veterinary services, regular treatment, and increased awareness [10,11]. Having sick or injured pets can be a cause of stress and worry for people, particularly if there are barriers in accessing animal health care [11,12]. A study of community members in rural and remote Aboriginal communities with limited access to animal health care recognised that parasites (such as mites, fleas, and heartworms) and gastrointestinal diseases (such as salmonella and giardia) are problems encountered in the dog population, as well as noise pollution, dog bites, and destructive behaviour [13]. Dog bites are common in remote communities in North Australia and can be due to large and unmanaged dog populations, causing both physical injury and psychological effects [14,15]. Dogs can also be carriers and reservoirs of zoonotic antimicrobial-resistant bacteria which is a significant public health concern [16].

There is increasing interest in addressing animal health care barriers and public health concerns in low-resourced settings using a One Health approach. One Health is a cross-disciplinary approach that considers the relationships between human, animal, and environmental health [17]. A One Health approach is a holistic approach to animal health and management, aligning with Aboriginal cultural and community contexts and connecting to Aboriginal ways of knowing, doing, and being. An advantage of One Health is the ability to improve wellbeing and health at a community level, rather than just one aspect of society [18]. Studies on dog management in First Nations communities internationally have concluded that strategies for addressing dog population management should involve community participation and consultation, fostering relationships, and support and engagement to be effective [13,19]. This is pertinent in Australian Aboriginal communities where dogs have a key role in cultural beliefs, families, and community life [12,20].

A remote community that faces barriers in accessing animal health care is Wadeye in the Northern Territory, Australia. Wadeye has a population of approximately 2280 people, 77% of whom are Aboriginal [21]. There is very limited access to animal health care and animal medicines with the closest veterinarian an approximately five-hour drive away in the greater Darwin region. A community-driven animal health and management program was implemented in Wadeye to address animal health and population concerns. The aim of this study was to evaluate this program to explore the application of a One Health approach to animal health and management in this setting.

## 2. Materials and Methods

### 2.1. Context

Due to the barriers in accessing animal health care, the Thamarrurr Development Corporation (TDC) Rangers, along with Animal Management in Rural and Remote Indigenous Communities (AMRRIC), implemented a companion animal health and management program, with support from the West Daly Regional Council. AMRRIC is a not-for-profit organisation that works to deliver veterinary care and animal-related education in rural and remote Aboriginal and Torres Strait Islander communities using a One Health approach.

### 2.2. Program Components

**Figure 1:**
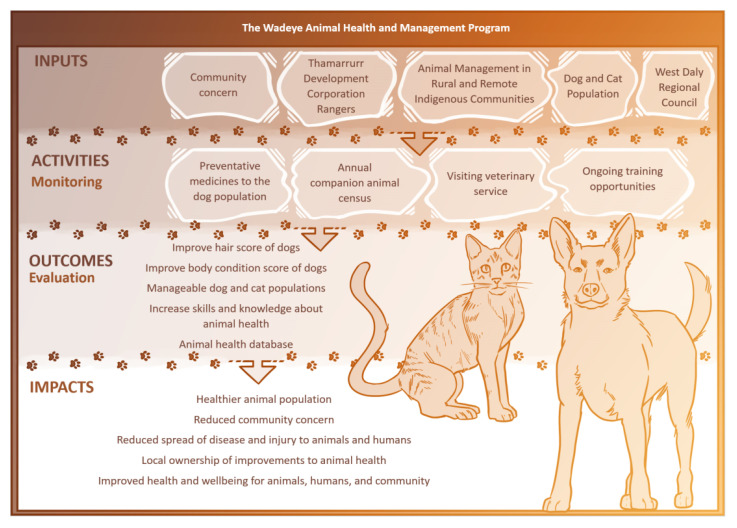
Logic model for the program.

The program consists of five main components including (see Figure 1):

- Co-developed animal health and management program
  ⚬ The TDC Rangers and AMRRIC started initial discussions in September 2016, followed by an initial veterinary visit and companion animal census in June 2017. The animal health and management program then began in June 2018 and is ongoing.
- Preventative medicines administered to the dog population
  ⚬ The TDC Rangers administered preventative medicines under veterinary direction, including ivermectin (a broad-spectrum anti-parasitic), to the dog population approximately every three months from February 2018 to June 2019. Afoxolaner (a broad-spectrum anti-parasitic) was administered to dogs that had an observed high burden of parasites. Additional preventative treatments (such as vaccination) were delivered by the visiting veterinary service when required.
- Annual companion animal census
  ⚬ AMRRIC adapted the census for use in Wadeye. The TDC Rangers, with support from AMRRIC, conducted three annual companion animal censuses from June 2017 to June 2019. The Rangers assist with translating the census questions to community members, as the majority of people do not speak English as a first language.
- AMRRIC visiting veterinary service
  ⚬ The visiting veterinary service, with assistance from the TDC Rangers, undertook weeklong visits to deliver desexing programs and undertake treatments and surgeries as needed. From June 2018 to June 2019, three veterinary visits occurred in Wadeye with 259 animals receiving treatment, 183 of which were surgically desexed.
- Ongoing training opportunities
  ⚬ The TDC Rangers undertake training, provided by AMRRIC staff and TDC’s Trainer, on recognizing common animal health concerns, preparing and administering preventative medicines, and collecting data.

### 2.3. Ethics

Ethics approval was provided by the Human Research Ethics Committee of the Northern Territory Department of Health and Menzies School of Health Research (2018-3176) and the Human Research Ethics Committee at the Australian National University (2018/588).

### 2.4. Study Design

Three data sources were used. A companion animal census was undertaken in June 2017, 2018, and 2019. The Rangers collected the data on a custom-designed AMRRIC app from local community households in Wadeye, with assistance from collaborating organisations. Data were captured at each household by observing the animals and asking the owners about the number of dogs and cats that reside there and their current health and reproductive status. The majority of animals were free-roaming, with mixed-breed medium-sized dogs most commonly seen.

The health outcomes of interest included desexing status (collected as desexed or can breed), body condition score (assessed using the Purina body condition score system and collected as skinny (score 1–3), normal weight (score 4–5), or overweight (score 6–9) [22]), and hair score (collected as good hair, some hair loss, or lots of hair loss). Age of animal was collected by age group including puppy/kitten (still suckling), young (weaning to almost full-grown size), adult (fully-grown size), and old (showing signs of aging or known to be over 5 years of age). Information on the size of the animals and additional health or welfare concerns that required veterinary attention was also captured, however, was not analysed in this study.

Ivermectin was administered at the same time as the census. Veterinary visit data were collected using the AMRRIC app on the number of animals seen and the type of treatments delivered. Dog bite data were collected at the health clinic in Wadeye for any patient that presented with a dog bite and aggregated quarterly from July 2016 to June 2019.

This was a pre-post design analysing three years of companion animal census data to assess animal health pre program implementation (2017) and over the initial 12 months of the program (2018 to 2019). Dog bite data were analysed over four years to assess presentations of dog bite patients to the health clinic, pre program implementation (2016 to 2018), and over the initial 12 months of the program (2018 to 2019). Veterinary visit data were also analysed to assess the extent and type of veterinary treatments delivered during the program (2018 to 2019).

An internationally used approach to design and measure companion animal management programs in communities is the International Companion Animal Management Coalition (ICAM) programs and indicators. The indicators focus on animal health, population numbers, and public health to assess the outcomes of the program on the community. Based on the ICAM Coalitions suggested indicators for assessing change, the following indicators and questions were developed [23] (see Table 1).

### 2.5. Variables

Census data were supplied line-listed by animal and identified by household for each census year. The main outcome variables included desexing status, which was coded as “can breed”, “desexed”, and “don’t know”. Body condition score was coded as “underweight”, “normal weight”, and “overweight”. Hair score was coded as “good hair”, “some hair loss”, and “lots of hair loss”. Exposure variables included the year which was coded as “2017”, “2018”, and “2019”. Species was coded as “cat” and “dog”. Variables were created to group the number of animals per household, with categories including “1–5”, “6–10”, “11–15”, and “16–24”. Age group was coded as “kitten”, “adult cat”, “puppy”, “young dog”, “adult dog”, and “old dog”. Sex was coded as “male”, “female”, and “don’t know”.

Dog bite data were supplied as the quarterly aggregated number of people presenting to the Wadeye health clinic for a dog bite, overall and by sex. Veterinary visit data were supplied as the aggregated number of animals that received each type of treatment at each veterinary visit.

Outcome variables were categorised into binary variables for regression analysis. For all outcomes, responses of “don’t know” were coded as “missing” and not included in the analysis. The regression analysis also excluded any animals missing age or sex. Body condition score was coded as “normal weight/overweight” (good body condition) versus “underweight”. Hair score was coded as “good hair” (good hair score) versus “any hair loss” (some and lots of hair loss combined). Desexing status was coded as “desexed” versus “not desexed”. Year was coded as “2018” and “2019” with 2017 coded as missing, with the exception of hair score which analysed “2017” and “2019”, with 2018 coded as missing due to a large amount of missing data.

### 2.6. Statistical Analysis

Statistical analysis was undertaken using Stata 15 and Excel.

Descriptive tables showing counts and percentages were used to summarise animal census data by year for total animal population and each animal health outcome variable. The number of animals per 100 people in Wadeye for three years was calculated using the Australian Bureau of Statistics (ABS) 2016 census population data [21]. The average number of animals per household was calculated using the number of animals divided by the number of households involved in the census each year. Stacked bar charts were created to visualise the distribution of each outcome by year. Veterinary visit data were analysed using descriptive tables broken down by the three veterinary visits and combined overall for a total over 12 months (June 2018–June 2019).

Regression analyses were used to quantify differences in outcomes from pre to post program implementation, comparing data from 2019 (post program implementation) to data from 2018 (pre program implementation). Prevalence ratios (PR) and corresponding 95% confidence intervals (CIs) were used to compare outcomes (body condition, hair score, and desexing status) in 2019 compared to 2018. In the case of the hair score, data from 2017 were used as the pre-intervention time point (instead of 2018) due to a high level of missing data on the outcome in 2018. All regression models are presented unadjusted and adjusted for age group and sex of the animals because they were potentially confounding variables. Adjusted results are presented in the text. A result was considered significant at a *p*-value of < 0.05.

Dog bites were presented as quarterly dog bites per 1000 population, by sex of person and overall. ABS 2016 census population data for Wadeye were used as the denominator to calculate crude rates. A time-series analysis was performed on total dog bites comparing July 2016–June 2018 (pre program implementation) to July 2018–June 2019 (the program period).

## 3. Results

### 3.1. Animal Population and Health Outcomes

From 2017 to 2019, the companion animal population decreased from 732 to 633. Across the years, dogs constituted the majority of pets (81–84%) in the community, with cats constituting the remaining 16–19%. From 2017 to 2019, there was a decrease in the number of dogs (598 to 532) and cats (134 to 101) owned as pets. There were 32.1 animals per 100 people in 2017 and 27.8 animals per 100 people in 2019. The average number of animals per household remained relatively stable, with 3.6 in 2017, 3.9 in 2018, and 3.7 in 2019 (see Table 2).

Across the years, less than half of the dog and cat population was desexed, with the percentage of desexed animals improving over time. The majority of dogs were of normal weight, with very few animals overweight and the underweight population improving over time. The majority of dogs had a good hair score in 2017 and 2019 (2018 data were not analysed due to the amount of missing data) (see Table 2). There were few households with large numbers of animals (>10), with the majority of households having 1–5 animals (77–82%) or 6–10 animals (15–23%) per household. Across the years, the majority of the dogs and cats in Wadeye were adults, with 70.8% of the dogs and 73.7% of the cats being adults in 2019. The percentage of old dogs was 3.1% in 2017 and 9.4% in 2019.

There was a 150% increase in the prevalence of desexed dogs from 2018 to 2019 (PR 2.50, 95%CI 1.70–3.70). Similarly, there was a 125% increase in the prevalence of desexed cats from 2018 to 2019 (PR 2.25, 95%CI 1.14–4.47). The prevalence of good body condition in dogs was 77% higher in 2019 compared to 2018 (PR 1.77, 95%CI 1.45–2.15). The prevalence of good hair score was 9% higher in 2019 compared to 2017 (PR 1.09, 95%CI 1.03–1.16) (see Table 3).

### 3.2. Dog Bites

The rate of clinical presentations to the health clinic for dog bites per quarter ranged from 1.3 to 7.0 per 1000 population between July 2016 and June 2019. The average quarterly rate pre program implementation was 4.7, and the average quarterly rate during the program was 4.2 dog bites per 1000 population. There was no difference in dog bites by sex of people presenting to the health clinic. Time-series analysis showed that the rate of dog bite presentations was increasing on average by 0.19 presentations per 1000 population (9.33 + 0.19 * Time) per quarter before the program was implemented. During program implementation, the rate of dog bites was decreasing on average by 0.20 presentations per 1000 population (11.20 − 0.20 * Time) each quarter. The difference in the slope of the two trends was not significant.

## 4. Discussion

From 2017 to 2019, the companion animal population decreased from 732 to 633. In 2019, there was an average of 3.7 animals per household in Wadeye, almost three times the national average (1.3 animals per household in 2016) [24]. Wadeye had a total of 27.8 animals per 100 people, with more dogs (23.3 dogs per 100 people) compared to the Australian average (20.0 dogs per 100 people). It is important to note that on average households have a higher number of people in Wadeye (6.1 people per household) compared to the Australian average (3.2 people per household); therefore, we can expect more animals per household [21]. There were substantially fewer cats in Wadeye (4.4 cats per 100 people) compared to the Australian average (16.0 cats per 100 people) [24]. Both animal and human population numbers are a snapshot and do not take into account changes to the populations over time. Wadeye is a large remote community; however, as this program focuses on maintaining animal population control and improving animal health, this program may also be suitable for communities with smaller animal populations.

The results show that after program implementation all animal health outcomes improved significantly. The prevalence of desexed dogs and cats more than doubled in 2019 compared to 2018. There was a 77% increase in the prevalence of good body condition in dogs in 2019 compared to 2018 and a 9% increase in the prevalence of good hair score in 2019 compared to 2017. There was also a decrease in the overall animal population size, an aging dog population, and fewer households with large numbers of animals. Due to the non-randomised, pragmatic nature of the evaluation, these improvements cannot be solely attributed to the program. However, the observed improvements were consistent with the hypothesised outcomes of the program, and the improvements occurred following program implementation. We therefore expect that at least some of the observed improvements can be attributed to the program itself.

Sustained animal health and management programs are important components in remote communities to keep animal populations healthy and manageable. Desexing animals can lead to a more manageable animal population and fewer animal-associated injuries [25]. Reaching a stable population is supported by desexing to counteract the rate of reproductive success in breeding females [10]. Dogs can be underweight and have hair loss due to high burdens of parasites and may also be underweight due to the effects of continual breeding, among other causes. Additionally, keeping animals healthy and reducing breeding can lead to an aging and manageable animal population with fewer animals per household. This may reduce the risk of communicable diseases transmitting to other animals and people [10]. Community-led approaches to addressing animal health concerns can be a feasible and sustainable option for controlling zoonoses and could be assessed by collecting human health data on zoonotic diseases affecting people in communities [18]. Increasing community awareness and local skills and knowledge can also assist in building effective animal management programs, with community ownership of the program beneficial [26].

There was no observed significant difference in dog bites pre to post program, overall and by sex; however, the dog bite findings were consistent with a decrease in the rate of dog bites in the community over the period of program implementation, compared to pre program implementation. The average quarterly rate pre program was 4.7 dog bite presentations per 1000 population, and the average quarterly rate during the program was 4.2 dog bites per 1000 population. The dog bite data relied on a dog bite injury being of sufficient severity to require medical attention; therefore, this analysis does not capture injuries that were not severe enough to require treatment.

To see programs such as this be sustainable and continue in communities, ongoing access to resources is needed. Building monitoring and evaluation capacity into program designs can produce evidence of the impact of the program, assess if the program is meeting community needs, and lead to increased support and funding [27,28]. Enhanced partnerships and collaboration between the animal, human, and environmental health sectors will assist in delivering a One Health approach [18]. Similarly, using evidence to influence the policy environment may lead to government-level support for One Health programs. The Northern Territory does not have companion animal management legislation, however, discussions are in place to enact this [29]. This means that currently there is no territory-wide legislation regarding companion animal management, and therefore, animal management is not consistently resourced or delivered between local government regions.

The strengths of this study include the inclusion of an annual census of the animal population before the program was developed, at the start of the program, and 12 months later, allowing a quantitative evaluation of the program. The community-driven component of this program was vital, with the involvement of community members likely to lead to a greater chance of the continuation of the program. The community-driven arrangement of the program allowed the Rangers to build on their skills and knowledge about animal health and management, leading to a higher likelihood of sustainability of the program. Additionally, this program had cooperation and support from multiple sectors including the local council, federal government, a not-for-profit animal health organisation, a Ranger group, and research scientists working in partnership and fostering a One Health approach.

The limitations of this study include the lack of completeness of the census data, as there is a substantial amount of missing data (including responses of “don’t know”) over the three years. In 2018, this was mainly due to technical issues. The prevalence of missing data was lower in 2019 than in previous years for most outcomes. This may be due to holding detailed refresher training with the Rangers, and the increasing experience with using the census app by delivering it annually. The analysis of dog bites had limitations as the data were supplied aggregated and the April–June 2018 quarter could not be separated into pre and post program implementation. There were also not enough time points to run an interrupted time-series analysis on the data. Collecting data on this outcome at additional time points would increase the power in conducting the interrupted time series analysis. This analysis could also be strengthened by collecting information about how the dog bites occurred, as dog bites may be in response to human behaviour and not related to the overpopulation of animals.

The inability to link animal health and human health from the same household limits the relationships we can draw between improvements in health outcomes [30]. Further consideration could be given on how to implement an enhanced One Health model in this setting to improve the health of all aspects of the community. This could be achieved through improving partnerships and communication between animal, human, and environmental health sectors and programs within communities [31]. Multi-disciplinary approaches to designing, implementing, and evaluating programs and the ability to collect integrated household-level data on animal and human health simultaneously could assist with this [18]. Future studies could consider additional components and indicators to assess community engagement with the program and assess if the program is meeting community needs [32].

## 5. Conclusions

This study showed the positive effects on animal health observed over the initial 12 months of a community-driven and One Health approach to animal health and management in a remote community. From June 2018 to June 2019, three veterinary visits were delivered with 259 animals receiving treatment. All animal health outcomes significantly improved and there was a decrease in the overall animal population, an aging dog population, and fewer households with large numbers of animals.

A One Health approach can be appropriate in this setting as it considers the relationships between animals, humans, and the environment, with improvement in animal health having the potential to improve human health and overall community wellbeing. Many programs such as this may not be supported long-term and rely on obtaining support and external funding to continue. The leadership of the community in implementing this program is vital to its sustainability, and through increasing skills and knowledge around animal health and management, this program has a greater chance of continuing. Future programs could consider incorporating a formal evaluation component to be able to produce and publish evidence of the impact of the program and assist in gaining continuing support.

## Figures and Tables

**Table 1:** Research questions, evaluation outcomes, indicators, and evaluation questions.

Research Questions	Intended Outcome of the Program	Indicator Measure of the Intended Outcome	Specific Evaluation Question
What is the state of animal health in Wadeye pre program implementation (2017)? How does the state of animal health change over the period of the program (2018 to 2019)?	Reduce or stabilise the animal population	Animals per 100 people Age group structure Breeding females Animals per household	To what extent were there changes in the companion animal population size and age distribution? To what extent were there changes to desexing status in the dog and cat population?
	Improve animal health	Body condition score of dogs Hair score of dogs	To what extent were there changes to animal health outcomes including dog body condition and hair score?
How many veterinary treatments are delivered over the program (2018 to 2019)?	Improve animal health care	Preventative and curative veterinary care Owner engagement	To what extent were veterinary care and animal medicines delivered? To what extent were households engaged in the program?
What is the rate of dog bite presentations to the health clinic pre program implementation (2016 to 2018)? How does the rate of dog bite presentations change over the program (2018 to 2019)?	Reduce risks to public health	Total number and rate of presentations to the clinic for dog bites	To what extent were there changes to the rate of presentations to the health clinic for a dog bite?

**Table 2:** Companion animal population in Wadeye by year.

Companion Animal Population in Wadeye by Year			
	2017	2018	2019
**Animal population**			
Species: % (n)			
Cat	18 (134)	19 (123)	16 (101)
Dog	82 (598)	81 (530)	84 (532)
Total	100 (732)	100 (653)	100 (633)
Animals per 100 people			
Cats per 100 people	5.9	5.4	4.4
Dogs per 100 people	26.2	23.3	23.3
Total animals per 100 people	32.1	28.6	27.8
Average animals per household			
Animals per household	3.6	3.9	3.7
**Animal health outcomes**			
Dogs desexed: %			
Desexed	28.6	18.5	37.0
Not desexed	57.4	54.9	46.1
Missing	14.1	26.6	16.9
Cats desexed: %			
Desexed	14.9	22.0	30.7
Not desexed	75.4	52.0	39.6
Missing	9.7	26.0	29.7
Dogs body condition score: %			
Overweight	1.2	0.8	3.8
Normal	62.2	48.7	76.7
Underweight	26.3	25.9	13.9
Missing	10.4	24.7	5.6
Dogs hair score: %			
Normal	69.4	29.1	80.3
Any hair loss	19.2	1.5	13.5
Missing	11.4	69.4	6.2

**Table 3:** Animal health outcomes in 2019 (post program implementation) compared to 2017/2018 (pre program implementation), unadjusted and adjusted for age group and sex.

Animal Health Outcomes in 2019 (Post Program Implementation) Compared to 2017/2018 (Pre Program Implementation), Unadjusted and Adjusted for Age Group and Sex										
	N	%	N	%	PR	95% CI	*p* Value	PR	95% CI	*p* Value
**Desexing status of dogs and cats pre and post program implementation (desexed versus not desexed)**										
Dogs	Desexed		Not desexed		Unadjusted			Adjusted for age group and sex		
2018	22	17.6	103	82.4	1			1		
2019	192	44.3	241	55.7	2.52	1.70–3.74	**<0.001**	2.50	1.70–3.70	**<0.001**
Cats	Desexed		Not desexed		Unadjusted			Adjusted for age group and sex		
2018	7	24.1	22	75.9	1			1		
2019	31	44.3	39	55.7	1.84	0.91–3.68	0.088	2.25	1.14–4.47	**0.020**
**Dogs body condition pre and post program implementation (normal weight/overweight versus underweight)**										
Dogs	Good body condition		Underweight		Unadjusted			Adjusted for age group and sex		
2018	53	48.2	57	51.8	1			1		
2019	419	85.0	74	15.0	1.76	1.45–2.15	**<0.001**	1.77	1.45–2.15	**<0.001**
**Dogs hair score pre and post program implementation (good hair versus any hair loss)**										
Dogs	Good hair score		Any hair loss		Unadjusted			Adjusted for age group and sex		
2017	408	78.6	111	21.4	1			1		
2019	422	85.6	71	14.4	1.09	1.03–1.15	**0.004**	1.09	1.03–1.16	**0.003**
